# Mechanical Loading Reshapes Mesenchymal Stem/Stromal Cell-Derived Extracellular Vesicle microRNA Cargo Toward Predicted Immunomodulatory and EV Cargo-Trafficking Pathways: An In Vitro Mechanobiology Study

**DOI:** 10.3390/biom16101393

**Published:** 2026-09-24

**Authors:** Mahammad Gardashli, Elena Della Bella, Manuel Herzog, Thomas M. Best, Martin J. Stoddart, Dimitrios Kouroupis

**Affiliations:** 1 Department of Orthopedics, UHealth Sports Medicine Institute, University of Miami Miller School of Medicine, Miami, FL 33146, USA; mahammad.gardashli@orlandohealth.com (M.G.); txb440@med.miami.edu (T.M.B.); 2 Diabetes Research Institute and Cell Transplant Center, University of Miami Miller School of Medicine, Miami, FL 33136, USA; 3 AO Research Institute Davos, 7270 Davos, Switzerland; elena.dellabella@aofoundation.org (E.D.B.); m.herzog29@pm.me (M.H.); martin.stoddart@aofoundation.org (M.J.S.)

**Keywords:** mesenchymal stem/stromal cells, extracellular vesicles, osteoarthritis, mechanical loading, mechanobiology, microRNA cargo, chondrogenic induction, oscillatory shear, macrophage polarization, biomanufacturing

## Abstract

Mesenchymal stem/stromal cell-derived extracellular vesicles (MSC-EVs) are emerging as cell-free therapeutic candidates for osteoarthritis, but most approaches treat the secretome as a passively collected product rather than a tunable biologic. Because joint loading regulates cartilage and synovial biology, we investigated whether biomechanical priming in a three-dimensional culture system could alter extracellular vesicle microRNA cargo toward cartilage- and immune-relevant regulatory programs. Bone marrow MSCs were seeded into macroporous polyurethane scaffolds within fibrinogen/thrombin hydrogels and cultured under four conditions: chondropermissive static (CP), chondropermissive uniaxial load (CPL), chondropermissive load plus oscillatory shear (CPLS), and chondrogenic static (CH). After 3 days, conditioned media were collected for extracellular vesicles isolation, particle concentration comparisons, RNA extraction, 166-microRNA profiling, and target/pathway analysis. EV preparations across all conditions demonstrated nanoscale particle distributions and strong CD63+/CD9+ positivity. CPLS-derived preparations achieved significantly higher particle abundance (2.9 × 10^8^ particles/mL) than all other conditions (*p* < 0.05). MicroRNA profiling identified a conserved 59-microRNA core shared across groups, with superimposed condition-specific remodeling. CH produced the broadest repertoire (138 microRNAs) with the strongest cell-cycle, senescence, and stress-regulatory signatures. Conversely, CPLS generated a highly focused profile (107 microRNAs) with a distinct membrane-directed cargo transport signature and the greatest overlap with the CH condition. Mechanically stimulated groups prominently engaged load-responsive MAPK and Wnt pathways, with CPLS demonstrating exceptionally strong predicted transforming growth factor-β (TGF-β) pathway engagement. Both CH and CPLS generated the strongest predicted M2 macrophage-associated regulatory signatures. Parental-cell validation confirmed that mechanical priming selectively modulated EV cargo sorting rather than passively mirroring intracellular abundance. Biochemical chondrogenic induction and mechanical priming generate distinct, non-interchangeable EV microRNA profiles. Combined compression and oscillatory shear increased particle yield and produced a focused cargo signature associated with predicted immunomodulatory, macrophage-related, and membrane-directed cargo-sorting programs. These findings support mechanobiological conditioning as a controllable upstream biomanufacturing variable for shaping MSC-EV miRNA cargo, while direct functional validation is required to determine therapeutic relevance in OA models.

## 1. Introduction

Osteoarthritis (OA) is a chronic and progressive musculoskeletal disorder characterized by articular cartilage degeneration, synovial inflammation, subchondral bone remodeling, pain, and functional decline. Affecting more than 300 million people worldwide, OA represents a major contributor to disability and health-care burden, particularly in aging populations. Despite its prevalence, current pharmacologic and surgical treatments remain largely palliative, providing symptom relief without reliably modifying the underlying disease process [1]. This therapeutic gap has driven growing interest in biologically based strategies that can address the inflammatory, catabolic, and regenerative components of OA pathogenesis.

Mesenchymal stem/stromal cells (MSCs) have emerged as promising candidates for regenerative and immunomodulatory approaches in OA. Although MSCs were initially investigated for their multilineage differentiation potential, their therapeutic effects are now largely attributed to paracrine mechanisms, including the release of cytokines, growth factors, lipid mediators, and extracellular vesicles that regulate local tissue and immune responses [2]. In the OA joint, macrophage phenotype is particularly relevant because classically activated M1 macrophages contribute to synovitis and cartilage degradation through mediators such as TNF-α and IL-6. In contrast, alternatively activated M2-like macrophages are associated with inflammation resolution, matrix remodeling, and tissue repair. MSCs and MSC-derived secretomes have been shown to influence macrophage polarization toward reparative phenotypes through multiple paracrine signals [3,4,5]. These findings support the concept that MSC-based therapies may act less through direct tissue replacement and more through modulation of the inflammatory joint microenvironment.

In this context, MSC-derived extracellular vesicles (EVs) have attracted increasing interest as cell-free therapeutic candidates for OA. EVs can transfer regulatory cargo, including microRNAs, long non-coding RNAs, proteins, and lipid mediators, and may reproduce key aspects of MSC paracrine activity while avoiding some limitations associated with live-cell transplantation, including variable engraftment, persistence, and safety concerns [6]. Preclinical OA studies have reported that MSC-EVs can suppress synovial inflammation, protect cartilage, modulate macrophage responses, and improve joint function [3,6,7,8,9,10,11,12,13].

However, most EV-based approaches still treat the MSC secretome as a passively collected product rather than a tunable therapeutic output. This is a critical limitation because EV yield, composition, and biological activity can vary substantially depending on donor source, culture conditions, priming strategy, and manufacturing parameters.

Mechanical cues represent one potentially important but underdeveloped regulator of MSC-EV production. Physiological joint motion exposes resident cells to coordinated compression, shear, and hydrostatic forces that are essential for cartilage homeostasis and mechanobiological signaling [14]. These loading environments influence MSC behavior, chondrogenic differentiation, inflammatory signaling, and extracellular matrix remodeling [15]. Importantly, we have previously shown that chondrogenic differentiation can be induced without the addition of exogenous TGF-β when MSCs are embedded in polyurethane-fibrin scaffolds that are exposed to daily mechanical load [16,17]. Emerging evidence suggests that mechanical conditioning can also alter the MSC secretome and EV output, including signals associated with inflammation resolution, tissue repair, and EV stability [18]. Therefore, mechanical priming may provide a rational upstream manufacturing strategy to better align MSC-EV cargo with the biomechanical and inflammatory environment of the OA joint.

Despite this rationale, the effects of defined mechanical loading on MSC-EV microRNA cargo remain incompletely understood. It is unclear whether biomechanical priming produces EV profiles that resemble biochemical chondrogenic induction, generate distinct mechanosensitive cargo states, or converge on shared immunomodulatory and chondroprotective regulatory pathways. Addressing this question is important for the development of standardized, mechanism-informed MSC-EV preparations for OA.

In this study, we investigated how defined biomechanical and biochemical culture conditions alter the microRNA cargo of bone marrow MSC-EVs generated in a three-dimensional scaffold-hydrogel culture system. We compared chondropermissive static culture, chondropermissive uniaxial compression, chondropermissive compression with oscillatory shear, and chondrogenic static induction. We then evaluated EV identity, particle abundance, microRNA repertoire, predicted target pathways, macrophage polarization-associated regulatory signatures, and selected parental MSC microRNA expression. We hypothesized that mechanical stimulation would remodel MSC-EV microRNA cargo in condition-specific ways and enrich predicted regulatory programs relevant to cartilage homeostasis and immunomodulation.

## 2. Materials and Methods

### 2.1. Study Design

This in vitro mechanobiology study evaluated whether defined biochemical and mechanical priming conditions alter the extracellular vesicle microRNA cargo of human bone marrow-derived MSCs. Cells from five anonymized human donors were expanded and cultured in three-dimensional polyurethane/fibrin scaffold constructs under chondropermissive static, chondrogenic static, chondropermissive uniaxial compression, or chondropermissive uniaxial compression plus oscillatory shear conditions. Conditioned media were collected for extracellular vesicle isolation and characterization, and scaffold-associated cells were collected for parental-cell microRNA analysis.

### 2.2. Cell Isolation and Culture/Isolation and Expansion

Vertebral bone marrow samples were collected from five donors (*n* = 5: male 71 y, male 61 y, female 73 y, female 69 y, male 73 y), undergoing spinal surgery at the Inselspital Bern, Switzerland, with written informed consent. The Swiss Human Research Act does not apply to fully anonymized samples, and a clarification of responsibility was obtained (Bern Req-2020-00141). All biological donors were treated as individual samples and were not pooled together for all experimentation described below.

Human bone marrow-derived MSCs were isolated as previously described [19]. For cell expansion, samples from all donors were seeded at a density of 3 × 10^3^ cells/cm^2^ in α-MEM (Gibco, Thermo Fisher, Zürich, Switzerland) with the addition of 10% MSC-qualified fetal bovine serum (FBS) (Corning, Corning, NY, USA), 100 U/mL penicillin, 100 mg/mL streptomycin (Gibco) and 5 ng/mL basic fibroblast growth factor (bFGF) (Fitzgerald Industries International, Concord, MA, USA) in T300 cm^2^ cell culture flasks (TPP Techno Plastic Products, Trasadingen, Switzerland). Cultures were kept in a 37 °C/5% CO_2_ humidified atmosphere and the medium was refreshed every second day. MSCs were passaged at a confluency of 80% until passage 3 (p3) for further usage.

### 2.3. MSCs Seeding in PU Scaffolds

Porous elastic polyurethane (PU) scaffolds (⌀ 8 mm, h 4 mm) and rings (inner ⌀ 8 mm, outer ⌀ 14 mm, h 4 mm) with a pore size of 90–300 μm, were fabricated as previously described [20]. PU scaffolds were soaked in chondropermissive medium (CP) and degassed for two hours in a vacuum chamber. The CP medium consisted of DMEM 4.5 g/L glucose (Gibco), 1% non-essential amino acids (Gibco), 100 U/mL penicillin, 100 mg/mL streptomycin (Gibco), 50 mg/mL L-Ascorbic Acid-2-phosphate (Sigma-Aldrich, Buchs, Switzerland), 1% ITS+ Premix (Corning) and 10^−7^ M dexamethasone (dexamethasone-cyclodextrin complex, Sigma-Aldrich).

MSCs at passage 3 were trypsinized, counted, resuspended in 50 mg/mL fibrinogen solution (Sigma-Aldrich) and added to the sterile lid of a 1.5 mL Eppendorf tube. An equal volume of a 2 U/mL thrombin solution (Sigma-Aldrich) was then mixed with the fibrinogen-cell solution, and the porous scaffold was pressed into the lid, as previously described [21]. Each construct contained 5 × 10^6^ cells, with a final fibrinogen and thrombin concentration of 25 mg/mL and 1 U/mL, respectively. After a 1 h incubation at 37 °C, cell-laden PU/fibrin scaffolds were placed inside a PU ring and transferred to a polyether ether ketone (PEEK) bioreactor holder (Figure 1).

Samples were divided into 4 groups (chondropermissive free swelling, chondrogenic free swelling, chondropermissive load, chondropermissive load + shear) and covered with cell culture medium. The chondropermissive, load and load + shear groups were cultured in chondropermissive (CP) medium as described earlier. The chondrogenic group was cultured in chondrogenic medium, consisting of CP medium with the addition 10 ng/mL recombinant human active TGF-β1. All samples were placed in 10 cm Petri dishes and incubated in a 37 °C, 5% CO_2_ humidified atmosphere.

### 2.4. Bioreactor Loading

Mechanical loading of cell-scaffold constructs was conducted using the bioreactor system as previously described [22]. Loading was performed for 1 h a day over 3 consecutive days, starting the day after seeding. The load group was exposed to dynamic compression at 1 Hz with a 10% strain on top of a 10% static strain, resulting in a maximum strain of 20%. In addition to compression, the load + shear group was exposed to compression and a simultaneous ball oscillation of ±25° at 1 Hz. Unloaded groups served as control and were considered free swelling. Exactly 24 h after the third loading, scaffolds were collected for RNA isolation. Cell culture medium was also collected, snap-frozen in liquid nitrogen, and stored at −80 °C until use. Culture medium was changed on day 2 (Table 1).

### 2.5. Isolating EVs from MSC-Conditioned Media Collected from the Bioreactor

EVs were isolated from MSC-conditioned medium from five donors by a stepwise ultracentrifugation method. Briefly, the media centrifuged for 2000× *g* for 10 min and ultracentrifuged for 120,000× *g* for 4 h using SW 40 Ti Swinging-Bucket Rotor for Optima XPN ultracentrifuge (Beckman Coulter, Brea, CA, USA). Pre-enriched EVs preparations were stored in DPBS at −80 °C.

### 2.6. Extracellular Vesicle Biophysical Characterization

#### 2.6.1. Nanoparticle Tracking Analysis (NTA)

The size distribution and concentration of EVs (*n* = 5 donors) were analyzed using a NanoSight NS300 system (Malvern Panalytical, Westborough, MA, USA) operated with NTA software version 3.4. Samples were diluted 1:10 in DPBS to a final volume of at least 1 mL and equilibrated to room temperature prior to measurement. Samples were manually loaded using a syringe until particles were visible in the field of view.

For each sample, five videos of 15 s each were recorded at an infusion rate of approximately 50 µL/min. Data were analyzed using default parameters: detection threshold set to 5, 30 frames captured per video, and a measurement time of 30 s per replicate. Only traced particles were included in the analysis. Settings were kept constant for all samples to ensure measurement consistency.

Results were reported as particle size distribution metrics and particle concentration in particles/mL. Each result represents the mean of five individual measurements per sample.

#### 2.6.2. Flow Cytometry for Surface Markers

EVs (*n* = 5 donors) were characterized by flow cytometry to validate the presence of exosome-enriched particles based on CD9 expression in CD63-captured vesicles. Samples were first incubated overnight at 4 °C on a rotator with human CD63 magnetic beads (Invitrogen, Carlsbad, CA, USA), using 200 µL of beads per 1 mL of EVs suspended in DPBS. Following incubation, samples were placed on a magnetic rack for 2 min. Supernatants were carefully removed without disturbing the beads, which were then resuspended in 200 µL of flow buffer (DPBS supplemented with 0.5% BSA and 2 mM EDTA) to prevent aggregation.

Samples were split into two tubes for immunostaining: one for antibody staining and one as unstained control. An amount of 5 µL of anti-human CD9-FITC antibody (Invitrogen, Carlsbad, CA, USA) was added to 200 µL of bead suspension. After gentle mixing, tubes were incubated for 15 min in the dark. Samples were washed twice with 5 mL of flow buffer, using magnetic separation to retain the beads after each wash. Beads were finally resuspended in 200 µL of flow buffer, and were transferred into a 96-well plate for acquisition.

Flow cytometry data were acquired using a CytoFLEX S cytometer (Beckman Coulter), collecting 20,000 events per sample. Data were analyzed using Kaluza Analysis Software v.2.4 (Beckman Coulter). Only CD9+ events within the CD63-gated population were considered indicative of exosome-enriched EVs.

#### 2.6.3. Transmission Electron Microscopy Imaging

To visualize the morphology and structural integrity of EVs, transmission electron microscopy (TEM) was performed. Samples were adsorbed onto carbon-coated copper grids for 30 min, rinsed in phosphate buffer, fixed with 2% glutaraldehyde, and washed with double-distilled water. Grids were then stained with 2% aqueous uranyl acetate for 5 min, protected from light, and stored overnight. Imaging was performed at 80 kV using a JEOL JEM-1400 transmission electron microscope (JEOL, Tokyo, Japan), and images were acquired using an AMT BioSprint 12 digital camera (Advanced Microscopy Techniques Corp., Woburn, MA, USA).

### 2.7. miRNA Expression Profiling in EVs

miRNAs were extracted from isolated EVs (*n* = 5 donors) using the Total Exosome RNA and Protein Isolation Kit (Thermo Fisher Scientific, St. Bend, OR, USA) following the manufacturer’s instructions. RNA concentration and purity were determined using a NanoDrop ND-1000 spectrophotometer (Thermo Fisher Scientific). A total of 1 µg of EV miRNA was utilized for first-strand cDNA synthesis with the All-in-One miRNA First-Strand cDNA Synthesis Kit (GeneCopoeia, Rockville, MD, USA). Predesigned human MSC exosome 166 miRNA qPCR arrays (GeneCopoeia) were performed using 1000 ng of cDNA per sample and processed with a StepOne real-time thermocycler (Applied Biosystems, LLC, Carlsbad, CA, USA). Data analysis was conducted using GeneCopoeia’s online data analysis system. Ct values above 34 and undetermined were excluded. Mean values were normalized to *SNORD47* which was selected for its stable expression between groups (<0.5 cycle difference), and expression levels were calculated using the 2^−ΔCt^ method. Relative expression to *SNORD47* more than 10^0^ was considered as threshold for highly present miRNAs as cargo. All biological donors were treated as individual samples and were not pooled together for miRNA cargo characterization.

To analyze putative miRNA interactomes, a network-centric visual analytics approach was applied using miRNet (https://www.mirnet.ca/, accessed on 10 January 2026). miRNA target gene data were obtained from the miRTarBase v8.0 database, and interactome network refinement was performed with a 2.0 betweenness cutoff. Data were visualized in a topological miRNA–gene interactome network using a force atlas layout and hypergeometric test algorithm.

miRDB online database (http://mirdb.org (accessed on 2 February 2026)) for prediction of functional miRNA targets has been used to correlate highly expressed target genes in macrophages with specific miRNAs identified by EV miRNA profiling. MirTarget prediction scores are in the range of 0–100% probability, and candidate transcripts with scores ≥ 50% are presented as predicted miRNA targets in miRDB.

### 2.8. Isolation of Total RNA from Scaffolds

Scaffolds from *n* = 5 donors were homogenized using a Mixer Mill MM 400 (Retsch GmbH, Haan, Germany) for 6 min at 25 Hz, into 2 mL Eppendorf tubes with 6 mm stainless steel metal balls and 1 mL TriReagent (Molecular Research Center, Inc., Cincinnati, OH, USA). The samples were centrifuged at 12,000 RCF for 10 min at 4 °C to remove PU scaffold residues. Total RNA was extracted with 1-bromo-3-chloropropane (Sigma-Aldrich). After phase separation, RNA was precipitated from the aqueous phase by adding 2-propanol (Sigma-Aldrich). The RNA pellet was washed with 75% EtOH and finally reconstituted with diethylpyrocarbonate (DEPC)-treated H2O. After incubation at 60 °C for 10 min, the total RNA concentration and purity was evaluated by analysis with the UV-Vis spectrophotometer NanoDrop 1000 (Thermo Fisher). RNA was stored at −80 °C until further use.

### 2.9. miRNA Expression Analysis in Parental MSCs

miRNA expression analysis of parental MSCs has been performed on five donors. TaqMan^TM^ Advanced miRNA cDNA Synthesis Kit (Thermo Fisher) was used for cDNA synthesis, followed by qPCR analysis using TaqManTM miRNA assays in combination with the TaqManTM Fast Advanced Master Mix (Thermo Fisher) on a QuantStudio 6 Pro Real-Time PCR system according to manufacturer’s instructions. Normalization of miRNA expression was performed using a global average approach [23] and results were expressed as 2^−ΔCt^.

### 2.10. Reactome Enrichment Analysis

To analyze putative miRNA interactomes, a network-centric visual analytics approach was applied using miRNet (https://www.mirnet.ca/, accessed on 10 January 2026). miRNA target gene data were obtained from the miRTarBase v8.0 database, and interactome network refinement was performed with a 2.0 betweenness cutoff. Data were visualized in a topological miRNA–gene interactome network using a force atlas layout and hypergeometric test algorithm. Reactome enrichment outputs were visualized as domain-specific tile plots using eGraph 2.0, with tile size representing the number of associated genes and color intensity representing −log10 (*p* value); pathways were organized into four functional domains: EV cargo trafficking/protein sorting, immunomodulation/inflammatory signaling, cell-fate/stress regulation, and mechanotransduction/growth-factor signaling.

### 2.11. Statistical Analysis

Normality was assessed using the Kolmogorov–Smirnov test. For normally distributed data, one-way or two-way ANOVA was used for multiple-group comparisons, as appropriate. For EV particle concentration comparisons, pre-planned pairwise comparisons between CPLS and each of the other culture conditions were performed using two-tailed Welch’s *t*-tests without correction. Statistical analyses were performed using GraphPad Prism v7.03 (GraphPad Software, San Diego, CA, USA). Statistical significance was set at *p* < 0.05.

## 3. Results

EV populations from all culture conditions demonstrated strong CD63+/CD9+ positivity by flow cytometry, ranging from 98.5% to 99.9% (Figure 2). NTA confirmed nanoscale particle distributions across all groups, with major peaks concentrated within the expected EV size range. As EV populations were derived from identical numbers of parental cells seeded per condition, the mean particle concentrations were compared side-by-side. Specifically, the mean particle concentrations were comparable between CP and CH conditions at 2.0 × 10^8^ particles/mL, lower in CPL at 1.5 × 10^8^ particles/mL, and highest in CPLS at 2.9 × 10^8^ particles/mL. CPLS-derived EV preparations had significantly higher particle abundance than CP, CPL, and CH preparations by Welch’s *t*-test, corresponding to a 1.45-fold increase relative to CP, a 1.93-fold increase relative to CPL, and a 1.45-fold increase relative to CH (CPLS vs. CP, *p* = 0.0036; CPLS vs. CPL, *p* = 0.00088; CPLS vs. CH, *p* = 0.0033).

Analysis across groups revealed that the chondropermissive static (CP) condition (Figure 3A) established a baseline library of 89 identified miRNAs, primarily dominated by hsa-miR-144-3p and hsa-miR-20b-5p. The introduction of compressive loading in the chondropermissive uniaxial load (CPL) group (Figure 3B) modestly expanded the library to 93 miRNAs but triggered a significant shift in high-abundance candidates, specifically identifying seven novel enriched species, with hsa-miR-525-5p being the highest concentrated miRNA from any group. Conversely, the chondrogenic static (CH) group (Figure 3C) yielded the highest diversity with 138 distinct miRNAs, despite demonstrating a highly concentrated enrichment profile where hsa-miR-146a and hsa-miR-450a-5p emerged as the only dominant species. Finally, the combination of mechanical stimuli in the chondropermissive uniaxial load + oscillatory shear (CPLS) group (Figure 3D) resulted in a library of 107 species, characterized by the most focused profile in the study with only a single high-abundance candidate, hsa-miR-107, remaining enriched.

Across all four domains, predicted EV-miRNA target enrichment showed both a shared regulatory core and condition-specific remodeling. In the EV cargo-trafficking/protein-sorting domain (Figure 4A), CPL and CH showed the deepest overlap among high-coverage transport pathways, including protein localization (CPL: 1060 genes; CH: 902 genes), establishment of protein localization (CPL: 853 genes; CH: 735 genes), protein transport (CPL: 826 genes; CH: 710 genes), and intracellular protein transport (CPL: 500 genes; CH: 427 genes). In contrast, CPLS showed a distinct membrane-directed targeting signal, with protein targeting to membrane enriched with 461 associated genes, compared with 116 genes in CPL and 102 genes in CH, while this pathway did not appear in CP.

The mechanotransduction/growth-factor panel (Figure 4B) showed that MAPK and Wnt-related programs remained particularly prominent in mechanically stimulated conditions. MAPK signaling had the highest gene count in CPL (177 genes), followed by CH (154 genes), CPLS (137 genes), and CP (131 genes). Signaling by Wnt was enriched in CPL (174 genes), CH (153 genes), and CPLS (135 genes), while the Wnt signaling pathway showed the strongest statistical enrichment in CPLS (−log10 *p* = 8.27) relative to CPL (−log10 *p* = 6.33), CH (−log10 *p* = 5.54), and CP (−log10 *p* = 4.33). TGF-beta-associated signaling also remained prominent, with TGF-beta signaling strongest in CPLS (62 genes; *p* = 4.43 × 10^−12^) and TGF-beta receptor complex signaling strongest in CH (65 genes; *p* = 7.18 × 10^−16^).

The immunomodulatory panel (Figure 5A) retained a strong shared immune signature, with broad Immune System enrichment highest by gene count in CPL (591 genes), followed by CH (511 genes) and CP (404 genes), and Innate Immune System enrichment similarly highest in CPL (365 genes), followed by CH (320 genes) and CP (253 genes). NF-kappaB/TLR-associated enrichment remained prominent across conditions. The strongest broad regulation of I-kappaB kinase/NF-kappaB gene count was observed in CP (604 genes), whereas CH showed stronger statistical enrichment for several NF-kappaB/TLR-associated subpathways.

The cell-fate/stress panel (Figure 5B) strongly supported CH as the dominant cell-cycle/stress-associated state. CH showed the strongest statistical enrichment for cellular responses to stress (*p* = 6.57 × 10^−28^; −log10 *p* = 27.18), Cell Cycle (323 genes; *p* = 4.84 × 10^−20^), Cell Cycle, Mitotic (274 genes; *p* = 5.36 × 10^−20^), Cellular Senescence (115 genes; *p* = 2.95 × 10^−18^), Oxidative Stress Induced Senescence (77 genes; *p* = 9.64 × 10^−17^), and regulation of cell cycle (468 genes; *p* = 2.99 × 10^−10^). CH also showed high gene coverage in G1 phase-associated pathways, including G1 phase (605 genes) and G1 phase of mitotic cell cycle (303 genes), supporting the interpretation that chondrogenic induction generates a strong cell-fate/stress-regulatory signature rather than a purely immunomodulatory profile.

We further evaluated the potential functional relevance of the identified miRNAs by examining their associations with macrophage polarization toward an anti-inflammatory M2 phenotype (Figure 6). As shown in Figure 6, the static CP and CH conditions are primarily characterized by miR-582-5p, let-7i-5p, and miR-20b-5p, whereas mechanical loading shifts the miRNA profile toward miR-23a-3p, miR-107, and miR-4286. Among the mechanically stimulated groups, CPLS exhibits the most balanced distribution of M2-associated miRNAs. To quantify these group-specific patterns, Table 2 summarizes the number of miRNA species predicted to regulate five key M2-associated domains: IL10, representing anti-inflammatory signaling; CD200R1, associated with immune suppression; CD163 and MRC1, established markers of M2 polarization; and NGF, which supports the M2 phenotype. Consistent with the patterns observed in Figure 6, CH and CPLS demonstrate the greatest overall breadth of M2-associated miRNA targeting, with 42 and 40 total miRNA–target associations, respectively. Thus, Figure 6 illustrates the distinct miRNA compositions underlying M2 polarization across the experimental conditions, while Table 2 quantitatively demonstrates the extent to which these profiles converge on key anti-inflammatory and M2-regulatory pathways.

Beyond these functional associations with M2 polarization, we next examined the overall distribution, overlap, and condition-specific uniqueness of the EV miRNA profiles across the four experimental groups (Figure 7). The Venn diagram highlights a core population of 59 shared miRNAs (38.3%) present across all four conditions, suggesting a conserved regenerative signature in MSC-EVs. The chondrogenic static (CH) group demonstrated the highest level of specialization with 16 unique miRNAs (10.4%), including species such as hsa-miR-450a-5p and hsa-miR-122-5p. In contrast, mechanical stimulation groups showed much lower unique counts, with the CPL and CPLS groups containing only four (2.6%) and two (1.3%) unique miRNAs, respectively. The two mechanically stimulated groups showed limited condition-specific overlap, with only one miRNA (0.6%) shared uniquely between CPL and CPLS. Notably, the greatest condition-specific overlap was observed between CPLS and the chondrogenic static condition, which shared 20 miRNAs (13%). This suggests that the addition of oscillatory shear may shift the MSC-EV miRNA profile toward a subset of chondrogenic-associated regulatory molecules.

We further investigated the relative expression of eight candidate miRNAs within the parental MSCs used for EV isolation across the four experimental groups (Figure 8). Significant differential expression was observed across several regulatory miRNAs depending on the induction environment. Notably, hsa-miR-23a-3p, identified as a major component of the mechanical loading EV signature, showed the greatest cellular upregulation in the CPL group compared with all other conditions (*p* < 0.0001). Similarly, hsa-miR-411-3p and hsa-miR-296-5p exhibited significant increases in the CPL group relative to the static CP and CH controls (*p* < 0.05 to *p* < 0.0001). In contrast, certain miRNAs associated with the baseline chondropermissive state, such as hsa-miR-146a-5p and hsa-let-7e-5p, showed significantly reduced expression following transition to the CH or CPLS environments. Other candidates, including hsa-let-7i-5p and hsa-miR-15b-5p, maintained relatively stable expression across all groups despite their high abundance in the resulting EVs.

## 4. Discussion

This study shows that mechanical stimulation reshapes the molecular composition of MSC-EVs in distinct ways. In our dataset, static chondrogenic induction produced the broadest miRNA repertoire, whereas combined loading and shear yielded a more focused profile, suggesting that mechanical cues bias EVs toward different functional states. By profiling a mixed EV population that likely includes exosomes and microvesicles, we capture a wider view of the MSC secretome yet also introduce vesicle subclass heterogeneity. That distinction matters because MISEV2023 recommends operational EV terminology when biogenesis is not directly demonstrated, and prior work in OA has shown that both exosomes and microvesicles can be chondroprotective. In contrast, exosomes alone may be more immunosuppressive than microvesicles in inflammatory arthritis [24,25,26].

The expanded Reactome analysis broadens this interpretation by showing that priming altered predicted EV-associated miRNA target programs across four regulatory layers: cargo trafficking/protein sorting, immunomodulation, cell-fate/stress regulation, and mechanotransduction/growth-factor signaling. This structure is important because it suggests that the effect of priming is not limited to changing inflammatory pathway enrichment, but may also involve predicted programs related to intracellular cargo handling and protein/RNA transport.

The chondropermissive static (CP) group appears to represent a baseline immunomodulatory state with both anti-inflammatory and pro-inflammatory features. Its most abundant miRNAs, miR-144-3p and miR-20b-5p, have both been linked to suppression of inflammatory injury and tissue damage in relevant disease models [27,28,29,30]. This interpretation is consistent with the Reactome profile of CP EV targets, which prominently featured I-kappaB kinase/NF-kappaB and TLR-related signaling, and is further supported by the presence of let-7i-5p and miR-106b, both of which have been associated with dampening inflammatory signaling in OA-related contexts [31,32]. At the same time, CP was not uniformly protective, as miR-301a-3p was also among the higher-abundance species and has been associated with NF-kappaB activation, Th17-skewed inflammation, and elevated TNF-alpha and IL-17A signaling [33,34]. Taken together, the CP group is best interpreted as a homeostatic immunoregulatory baseline rather than a purely anti-inflammatory state.

The CH group (chondrogenic static) showed the greatest amount of distinct miRNA based on Figure 7, yet its highly abundant cargo was concentrated into only two dominant species, miR-146a-5p and miR-450a-5p. The literature on miR-146a-5p is mixed, with studies linking it both to OA-related injury and to chondroprotective effects depending on model and signaling context [35,36,37,38,39]. In contrast, miR-450a-5p has been reported to attenuate apoptosis, inflammation, and ECM degradation in cartilage injury models [40]. The CH group also contained the largest number of unique miRNAs (Figure 7), including several linked to chondrogenic differentiation and chondroprotective behavior, such as miR-30a-5p, miR-181a-3p, and miR-127-3p [41,42,43,44,45]. However, not all CH-specific species were clearly beneficial, as miR-34a-5p has been associated with chondrocyte apoptosis, senescence, and reduced proliferation [46]. This mixed profile aligns well with the Reactome enrichment pattern, where CH showed the strongest cell-cycle and stress-associated signature across multiple related pathways, including cellular responses to stress (*p* = 6.57 × 10^−28^), cell cycle (323 genes; *p* = 4.84 × 10^−20^), mitotic (274 genes; *p* = 5.36 × 10^−20^), cellular senescence (115 genes; *p* = 2.95 × 10^−18^), oxidative stress-induced senescence (77 genes; *p* = 9.64 × 10^−17^), regulation of cell cycle (468 genes; *p* = 2.99 × 10^−10^), G1 phase (605 genes), and G1 phase of mitotic cell cycle (303 genes). Overall, the CH EV-miRNA cargo appears functionally concentrated in chondrogenic, cell-fate, cell-cycle, senescence, and stress-regulatory programs, while still retaining immunomodulatory features.

Among the four conditions, CPL (chondropermissive uniaxial load) contained the largest set of highly expressed miRNAs, led by miR-525-5p. Prior work has linked miR-525-5p to disease progression through the GDF5 axis. Suppression of this miRNA has been associated with reduced oxidative stress, NF-kappaB signaling, and matrix degradation in OA chondrocytes [47,48,49]. Other abundant CPL-associated candidates, including miR-802 and miR-146a-5p, also support an injury and immunomodulatory interpretation. miR-802 has been linked to metabolic inflammation, macrophage modulation, and steatohepatitis, suggesting broader inflammatory relevance beyond cartilage-specific pathways. Additionally, miR-146a-5p has been repeatedly implicated in OA-related cartilage homeostasis, IL-1β-induced chondrocyte inflammation/apoptosis, SDF-1/CXCR4 signaling, autophagy and extracellular matrix degradation. However, because miR-146a-5p has been reported to exert both protective and disease-promoting effects depending on the model and signaling context, its enrichment should be interpreted as evidence of injury signaling rather than a uniformly pathogenic or therapeutic effect [35,36,37,38,39,50,51]. The presence of miR-218-5p among CPL-unique miRNAs further supports this pattern, as it has been linked to worsened chondrocyte apoptosis and synovial inflammation [52]. Overall, CPL EVs suggest that uniaxial loading broadens the highly expressed miRNA cargo and reinforces an immunomodulatory, injury-responsive signaling profile under chondropermissive conditions.

The EV cargo-trafficking/protein-sorting panel indicates that CPL and CH share deep overlap among the highest-coverage cargo-handling pathways. CPL and CH were both strongly enriched for protein localization (1060 and 902 genes, respectively), establishment of protein localization (853 and 735 genes), protein transport (826 and 710 genes), and intracellular protein transport (500 and 427 genes). This overlap suggests that uniaxial compression and biochemical chondrogenic induction may converge on broad protein localization/transport-associated regulatory programs.

In contrast, the CPLS group (chondropermissive uniaxial load with oscillatory shear) produced the most focused high-abundance profile in the study, with miR-107 as the single dominant candidate. miR-107 has been associated with reduced pyroptosis, lower matrix degradation, and improved chondrocyte survival [53]. CPLS also uniquely expressed miR-335-5p, a miRNA whose reported effects appear highly context- and stimulus-dependent. Across prior studies, miR-335-5p has been linked to chondrogenesis, reduced OA-associated chondrocyte inflammation through autophagy activation, donor-dependent regulation of collagen deposition during chondrogenic commitment, and cartilage degeneration with features of senescence and DNA damage under mechanical overload [54,55,56,57]. Therefore, miR-335-5p enrichment in CPLS should be interpreted as evidence of a mechanically sensitive regulatory signal related to chondrocyte development and cartilage homeostasis, rather than as a uniformly anabolic or pathogenic marker. The other CPLS-unique miRNA, miR-363-3p, has been associated with impaired chondrocyte proliferation and survival in inflammatory settings [58]. Notably, this focused cargo profile was accompanied by the highest EV particle abundance among all groups, with CPLS-derived EV preparations showing significantly higher particle concentrations than CP, CPL, and CH based on Welch’s *t*-test analysis. This suggests that CPLS may be distinct not only because it increased EV particle abundance, but also because its Reactome enrichment analysis was associated with a more membrane-directed predicted cargo-transport signature, linking mechanical shear to both EV yield and predicted cargo organization. This finding is consistent with prior work showing that mechanical forces can stimulate EV production, supporting the idea that combined loading and shear may influence both EV particle yield and cargo organization [59]. Overall, CPLS EVs suggest that combined uniaxial loading and oscillatory shear produces a focused miRNA profile related to chondrocyte survival, cartilage homeostasis, and injury-responsive adaptation.

Within the Figure 4 cargo-trafficking domain, CPLS was distinguished by a strong membrane-directed protein targeting signal. Protein targeting to membrane involved 461 genes in CPLS compared with 116 genes in CPL and 102 genes in CH, while it did not emerge in CP in the curated panel. This pattern supports the interpretation that combined compression and oscillatory shear may not simply broaden protein transport programs but may bias toward membrane-directed cargo localization and transport-related regulation.

Importantly, the mechanically stimulated groups, particularly CPL and CPLS, retained a prominent mechanosensitive signaling profile. MAPK signaling showed the highest gene count in CPL (177 genes), followed by CH (154 genes), CPLS (137 genes), and CP (131 genes), supporting the interpretation that uniaxial compression strongly engages MAPK-associated regulatory networks. Wnt-associated signaling also remained elevated in CH, CPL, and CPLS. Signaling by Wnt involved 174 genes in CPL, 153 genes in CH, and 135 genes in CPLS, while the Wnt signaling pathway showed the strongest statistical enrichment in CPLS (−log10 *p* = 8.27) relative to CPL (−log10 *p* = 6.33), CH (−log10 *p* = 5.54), and CP (−log10 *p* = 4.33). Together, these findings support the view that mechanical stimulation reshapes EV-miRNA cargo toward known load-responsive MAPK/Wnt-associated signaling pathways [60,61,62,63,64], while also showing partial overlap with biochemical chondrogenic induction. Fitzgerald et al. showed that shear- and compression-induced chondrocyte transcription requires MAPK activation, identifying MAPK as a central conduit by which cartilage converts physical loading into biological responses [61]. Similarly, Thomas et al. demonstrated that physiological tensile strain modulates Wnt/β-catenin signaling in articular chondrocytes, while also showing that Wnt activity in cartilage is highly context dependent [62]. TGF-β-related signaling was also retained across conditions in the Reactome pathway enrichment analysis, although this should be interpreted as pathway-level enrichment rather than direct targeting of TGF-β itself. In Figure 5, direct miRNA associations with the TGF-β regulatory domain were observed in CH, CPL, and CPLS, whereas the selected CP miRNAs did not directly overlap with known TGF-β-associated miRNAs. In contrast, the broader Reactome analysis in Figure 4 showed TGF-β-related pathway enrichment across conditions, with TGF-β signaling particularly strong in CPLS (62 genes; *p* = 4.43 × 10^−12^) and TGF-β receptor complex signaling strongest in CH (65 genes; *p* = 7.18 × 10^−16^). This distinction suggests that biochemical induction and mechanical stimulation may converge on TGF-β-associated regulatory programs through different miRNA architectures and pathway-level mechanisms. This interpretation is consistent with prior work by Li et al., who demonstrated that mechanical loading, including uniaxial and oscillatory loading, promotes hMSC chondrogenesis by increasing TGF-β gene expression and protein synthesis [65]. This broader framework is reinforced by Cherifi et al., who reviewed Wnt and TGF-β as major osteoarthritis-relevant signaling hubs in cartilage biology [63], and by Davidson et al. who emphasized that TGF-β is crucial for cartilage maintenance but context dependent in joint pathology [64]. Together, these findings support a model in which biochemical and mechanical priming converge on cartilage-relevant TGF-β/MAPK/Wnt signaling axes, while differing in the specific miRNA architecture and pathway intensity.

A central finding of this study is that different priming conditions can converge on related immunoregulatory pathways through different molecular architectures. Although CH and CPLS were clearly distinct in repertoire size, dominant miRNA composition, and degree of uniqueness, both showed prominent predicted targeting of genes associated with M2 macrophage regulatory programs. This is particularly notable in Table 2, where CH and CPLS had the highest total numbers of predicted M2-related miRNA hits, and CPLS showed stronger predicted targeting of IL10 and MRC1 than the CP baseline. As miRNAs typically suppress target gene expression, these findings should be interpreted as evidence of altered post-transcriptional regulation of macrophage-polarization pathways rather than direct evidence of increased IL10 or MRC1 expression. This observation is consistent with prior OA work showing that BM MSC-EVs can reduce synovial macrophage infiltration, suppress M1 polarization, increase M2 markers, and elevate IL-10 [66], but functional validation would be required to determine whether the predicted miRNA targeting observed here produces similar immunomodulatory effects. Overall, these findings support a network-based view of EV potency, in which different cargo configurations may converge on similar immunoregulatory pathways rather than depending on one canonical miRNA alone.

The Venn analysis further strengthens this interpretation by showing that all four groups retained a shared core of 59 miRNAs, while the condition-specific layer varied substantially. CH displayed the greatest specialization, whereas CPL and especially CPLS contained far fewer unique miRNAs. At the same time, the overlap between the two mechanically stimulated groups suggests that loading and shear recruit a partially shared mechanosensitive regulatory subset. Together, these findings suggest that priming modifies a shared core EV miRNA profile in condition-specific ways, rather than generating completely distinct EV cargo profiles.

Our parental-cell validation data also support the idea that EV loading is selective rather than a passive mirror of intracellular abundance. Some highly abundant EV miRNAs remained relatively stable in the parent MSCs, whereas others changed strongly with culture conditions, particularly under loading. The marked cellular upregulation of miR-23a-3p, miR-411-3p, and miR-296-5p in the CPL group, together with the relative stability of other EV-associated miRNAs, argues against a simple bulk-transfer model. This is consistent with recent profiling work showing that MSC-EVs carry selectively packaged core miRNA signatures that are not identical to those of their donor cells [67]. Thus, both biochemical and mechanical priming likely influence not only the intracellular transcriptional state but also regulated EV cargo sorting.

From a translational perspective, these findings suggest that mechanical priming may be an important unrecognized biomanufacturing variable, not just a minor culture condition. Recent work has argued that the mechanical microenvironment can influence EV yield, cargo selection, and therapeutic output [68]. Our data support this idea by showing that defined mechanical inputs can produce distinct EV miRNA profiles with interpretable biological patterns: CH produced a broad chondrogenic cell-cycle/stress-regulatory state, whereas CPLS produced a narrower but high-yield mechanically conditioned profile with prominent membrane-directed protein targeting and TGF-β/Wnt/MAPK-associated pathway enrichment.

Several limitations should be considered. Because our pathway, target, and interactome analyses are prediction-based, they identify plausible regulatory programs rather than confirmed causal mechanisms. As we profiled a mixed EV population, we also cannot assign specific functions to exosomes and microvesicles separately. In addition, much of the miRNA literature remains context dependent and, in some cases, comes from non-cartilage inflammatory systems, so direct translation to OA biology should be interpreted cautiously. Finally, although the macrophage-targeting results are compelling, direct co-culture and in vivo validation are still needed to determine whether CH- or CPLS-derived EVs outperform other preparations in promoting immunomodulatory macrophage responses and protecting cartilage under inflammatory stress. Future work should therefore combine vesicle subfractionation, uptake studies, macrophage polarization assays, cartilage rescue models, and standardized potency testing. Nevertheless, the current findings suggest that biochemical chondrogenesis and mechanical priming do not produce identical EV miRNA profiles. Instead, each condition generates distinct cargo patterns that may inform the development of indication-specific MSC-EV therapeutics for OA.

## 5. Conclusions

Biochemical and mechanical priming did not generate interchangeable MSC-EVs; instead, they produced distinct miRNA cargo profiles with different predicted therapeutic implications. Chondrogenic static induction produced the broadest and most specialized miRNA repertoire with prominent predicted cell-cycle, senescence, and stress-regulatory signatures; whereas, combined uniaxial compression and oscillatory shear produced the highest EV particle abundance and a more focused miRNA profile associated with membrane-directed cargo trafficking and predicted immunomodulatory/macrophage-related regulation. These findings suggest that EV potency is not defined by cargo quantity alone, but also by how priming conditions reshape EV miRNA cargo toward specific regulatory programs. Together, our preliminary data suggest that mechanobiological conditioning could be a controllable upstream biomanufacturing parameter to develop more standardized, mechanism-informed MSC-EV therapeutics for OA. Our proposed hypothetical blueprint for mechanobiological programming of MSC-EV is described in Figure 9.

## Figures and Tables

**Figure 1:**
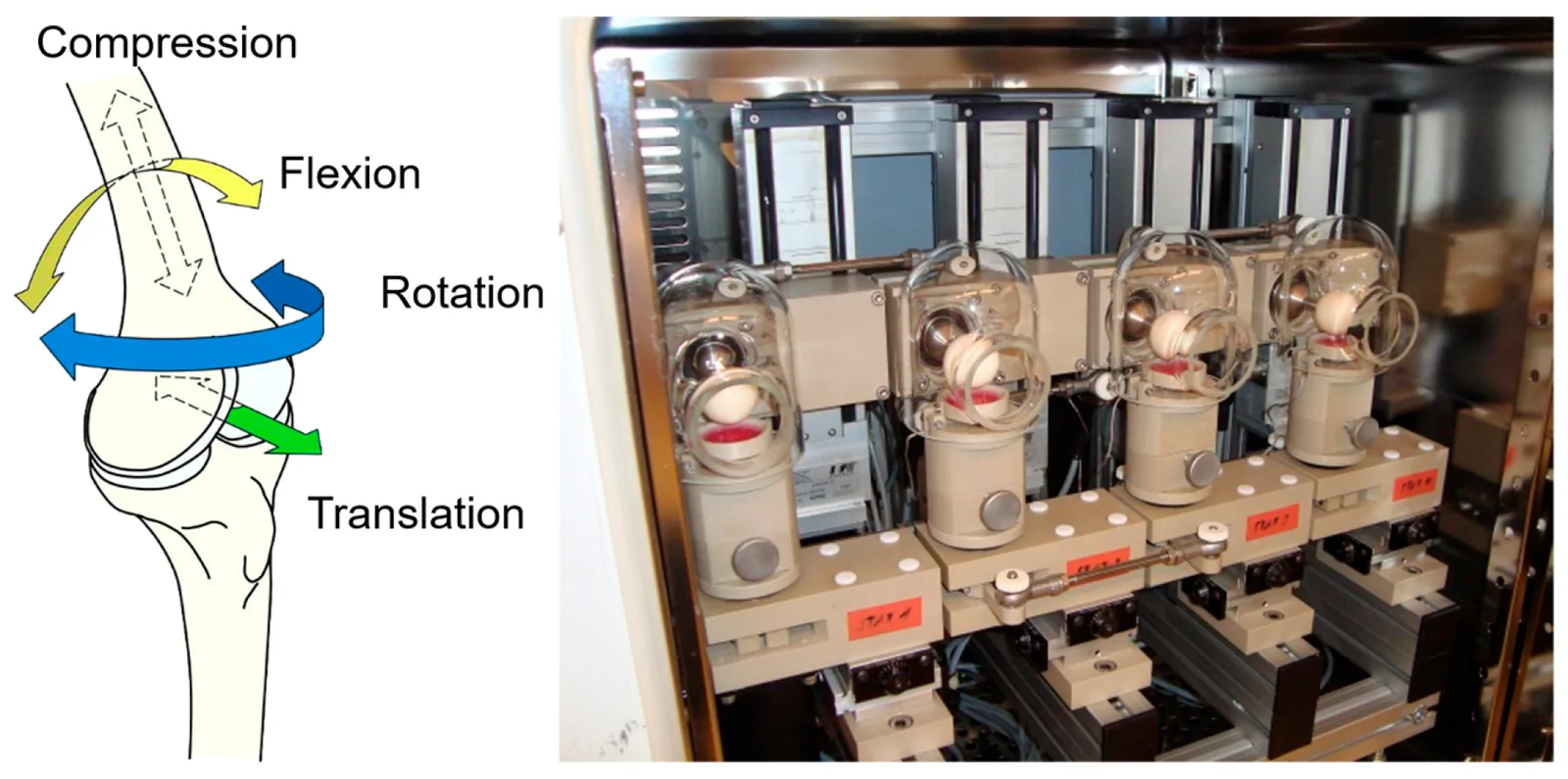
**Bioreactor loading system set-up.** Bioreactor system mimics the physiological joint biomechanical environment.

**Figure 2:**
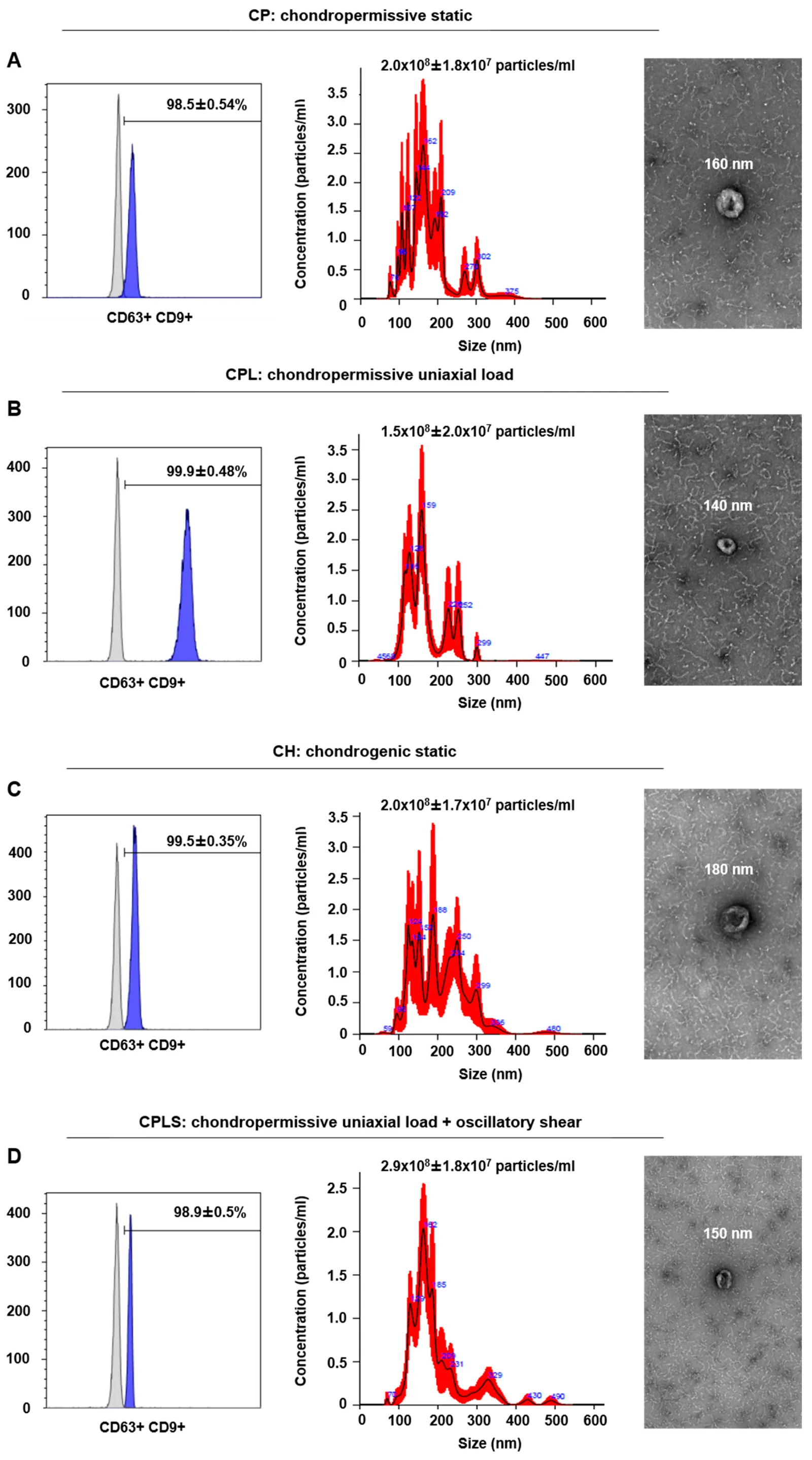
**EV biophysical characterization.** EVs isolated from CP (**A**), CPL (**B**), CH (**C**), and CPLS (**D**) culture conditions showed high CD63+/CD9+ positivity by flow cytometry and nanoscale particle distributions by NTA, with particle concentrations ranging from 1.5 × 10^8^ to 2.9 × 10^8^ particles/mL. Transmission electron microscopy reveals spherical, membrane-bound vesicles with intact morphology in all conditions. The EVs appear well-dispersed, with minimal aggregation, and are visualized using negative staining.

**Figure 3:**
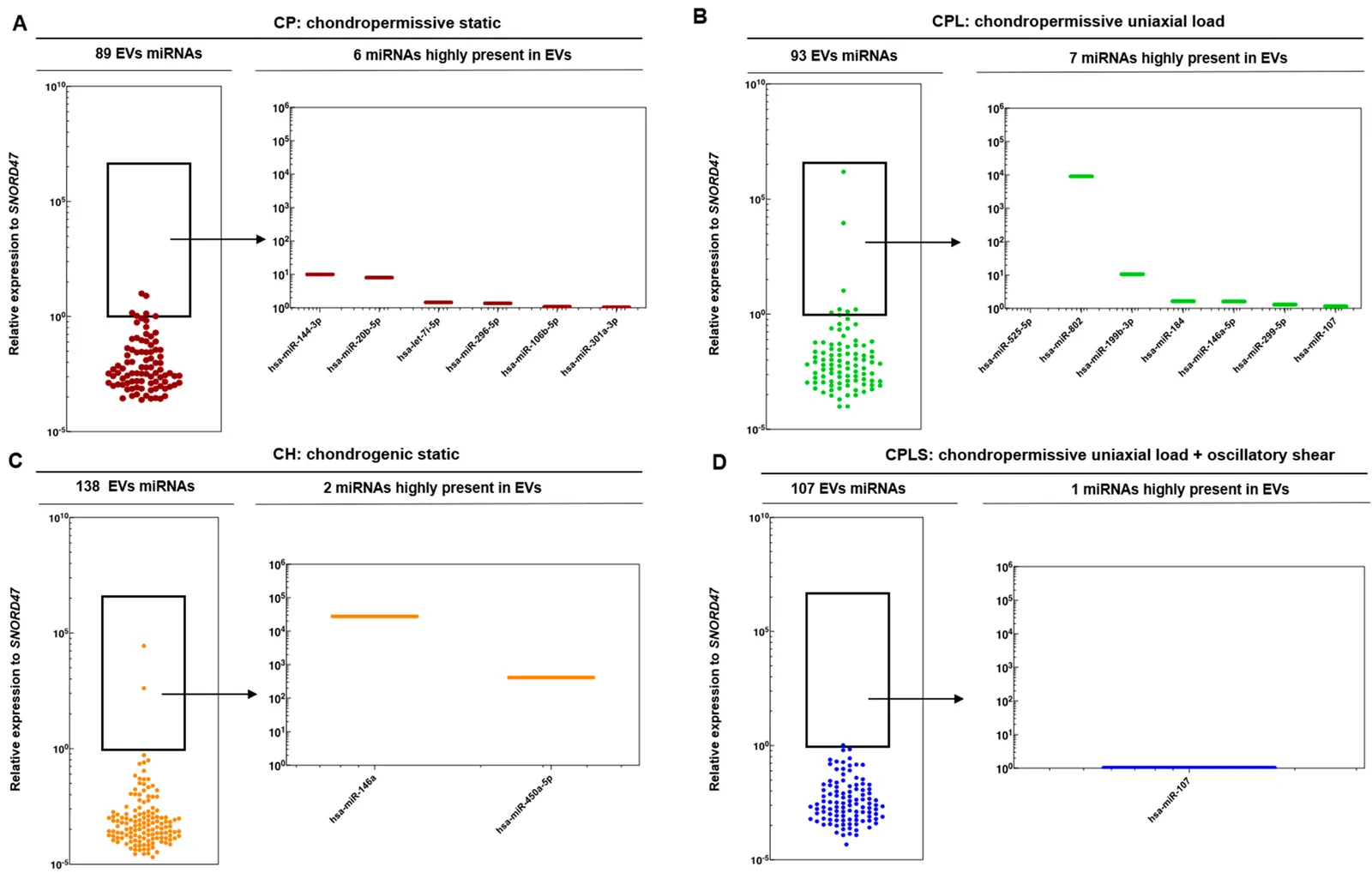
**Comparative profile of miRNA content in MSC-EVs across culture conditions.** This figure illustrates a quantitative comparison of the miRNA cargo within EVs isolated from MSCs cultured under four distinct loading conditions (Red (**A**) = chondropermissive static; green (**B**) = chondropermissive uniaxial load; orange (**C**) = chondrogenic static; and blue (**D**) = chondropermissive uniaxial load with oscillatory shear). Each panel displays a global dot-plot visualization of total detected miRNAs (left) and an expanded view identifying the specific, high-abundance miRNA candidates (right). The threshold for high-abundant miRNAs was set to >10^0^. Expression levels were calculated using the 2^−ΔCt^ method and normalized to the most stable HKG, *SNORD47*.

**Figure 4:**
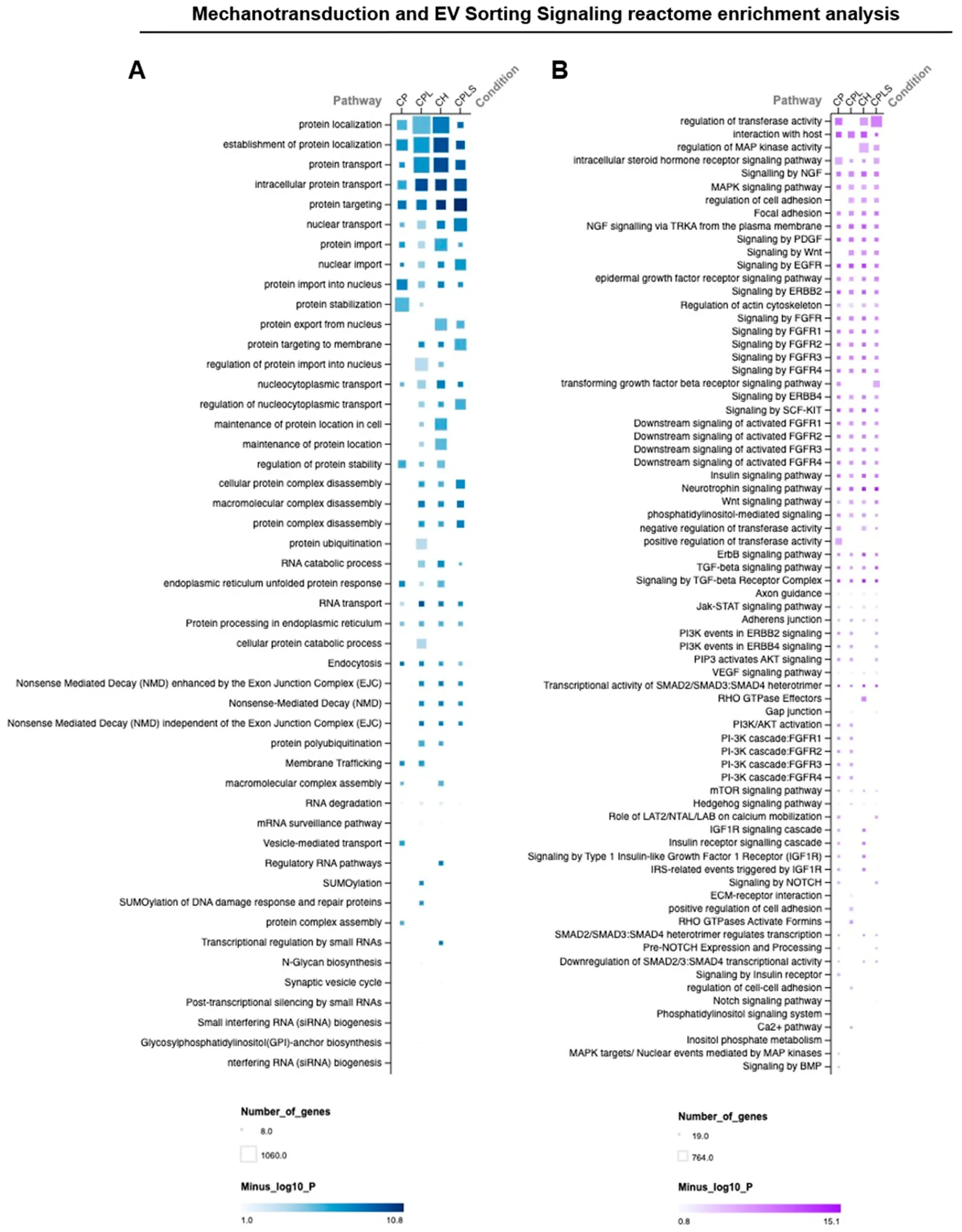
**Functional pathway and gene enrichment analysis of EV-associated miRNA targets across two regulatory domains**. The dot plots summarize enriched Reactome pathways predicted for EV-associated miRNA gene targets across CP, CPL, CH, and CPLS. Pathways are organized into two functional domains: (**A**) EV cargo trafficking/protein sorting and (**B**) mechanotransduction/growth-factor signaling. Square size represents the number of associated genes, and color intensity represents −log10 (*p* value). CPL and CH show substantial overlap in protein localization and protein transport pathways, whereas CPLS demonstrates a more distinct membrane-directed protein targeting signal. Mechanically stimulated conditions show prominent enrichment of MAPK/Wnt-associated signaling with condition-specific differences in TGF-β, neurotrophin, and growth-factor pathway intensity.

**Figure 5:**
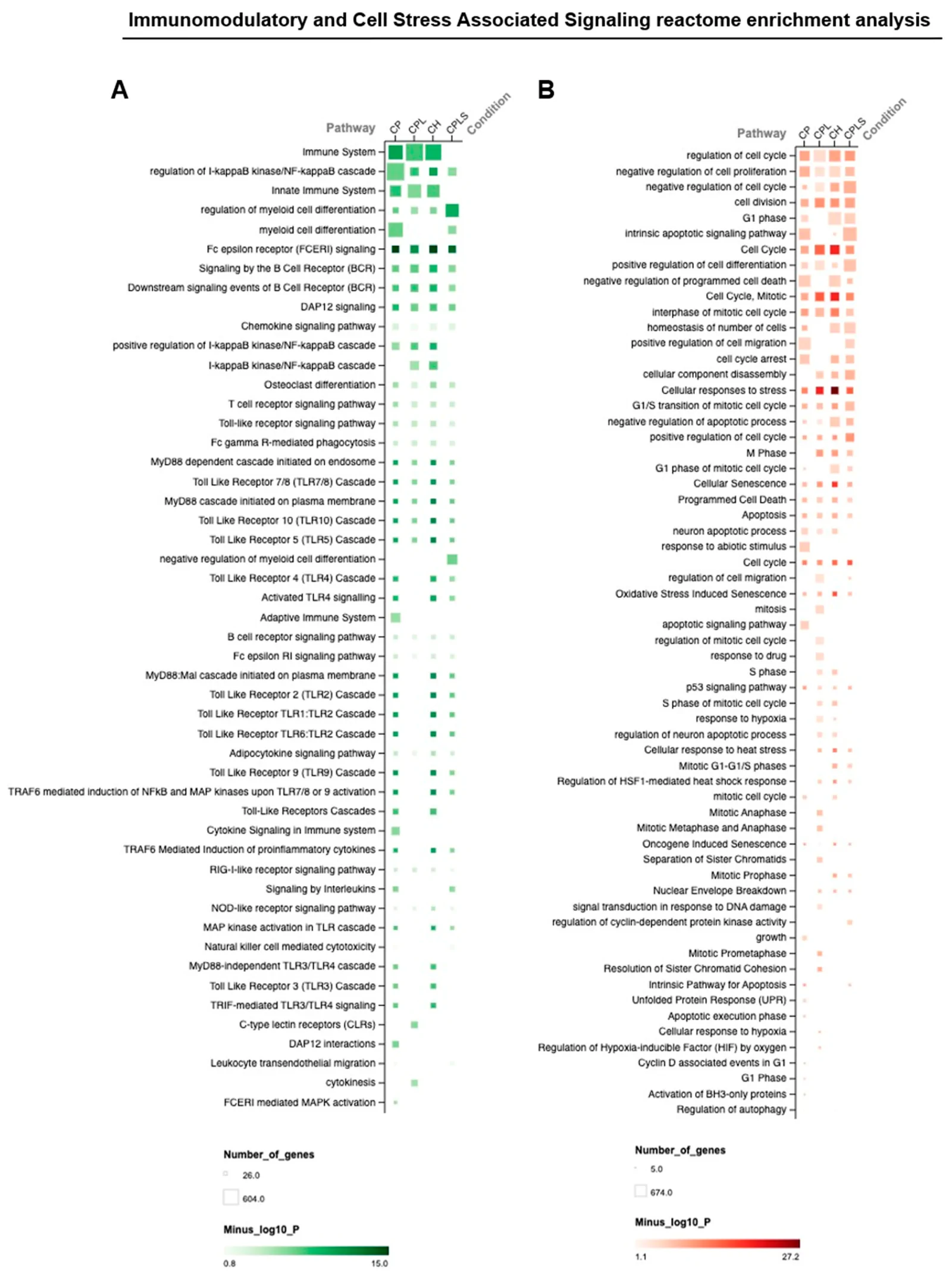
**Functional pathway and gene enrichment analysis of EV-associated miRNA targets across two regulatory domains**. The dot plots summarize enriched Reactome pathways predicted for EV-associated miRNA gene targets across CP, CPL, CH, and CPLS. Pathways are organized into four functional domains: (**A**) immunomodulatory panel and (**B**) cell-fate/stress panel. Square size represents the number of associated genes, and color intensity represents −log10 (*p* value). Across both domains, CPL and CH show the most consistent shared enrichment, with overlapping innate immune/TLR–NF-κB signaling in the immunomodulatory panel and shared cell-cycle, apoptosis, and stress-response enrichment in the cell-stress panel; whereas, CPLS shows a more distinct immunomodulatory profile marked by prominent myeloid differentiation-related enrichment and CP/CPLS display more condition-specific stress signatures.

**Figure 6:**
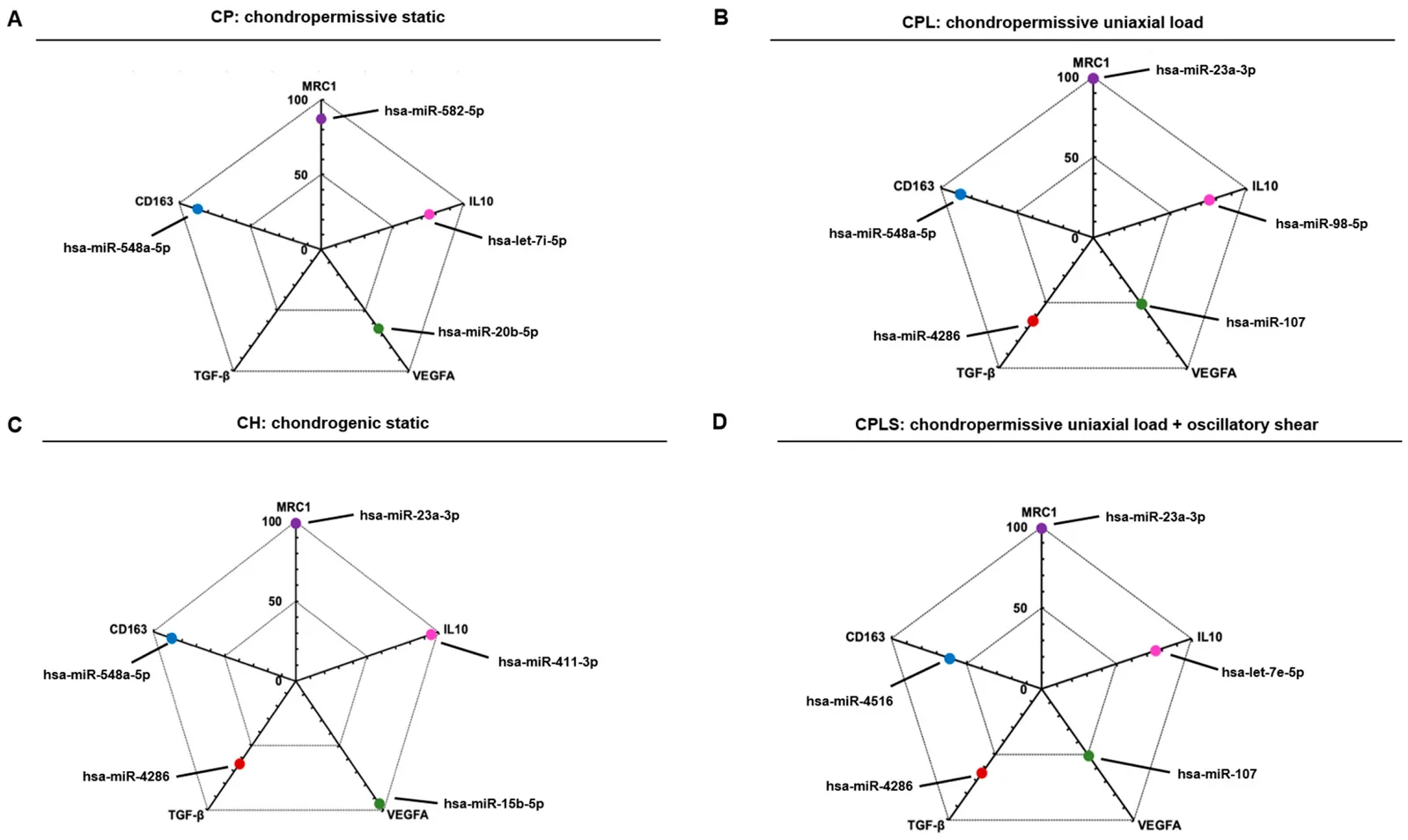
**Strength of miRNA association with key regulatory genes in M2 macrophage polarization.** Plots illustrate the association strength between highly abundant miRNAs and five key regulatory genes involved in M1-to-M2 macrophage polarization: MRC1, IL10, VEGFA, TGF-β, and CD163. Across CP (**A**), CPL (**B**), CH (**C**), and CPLS (**D**), MRC1/CD206 and CD163 are consistently strongly targeted, while the miRNAs driving these effects shift by culture condition.

**Figure 7:**
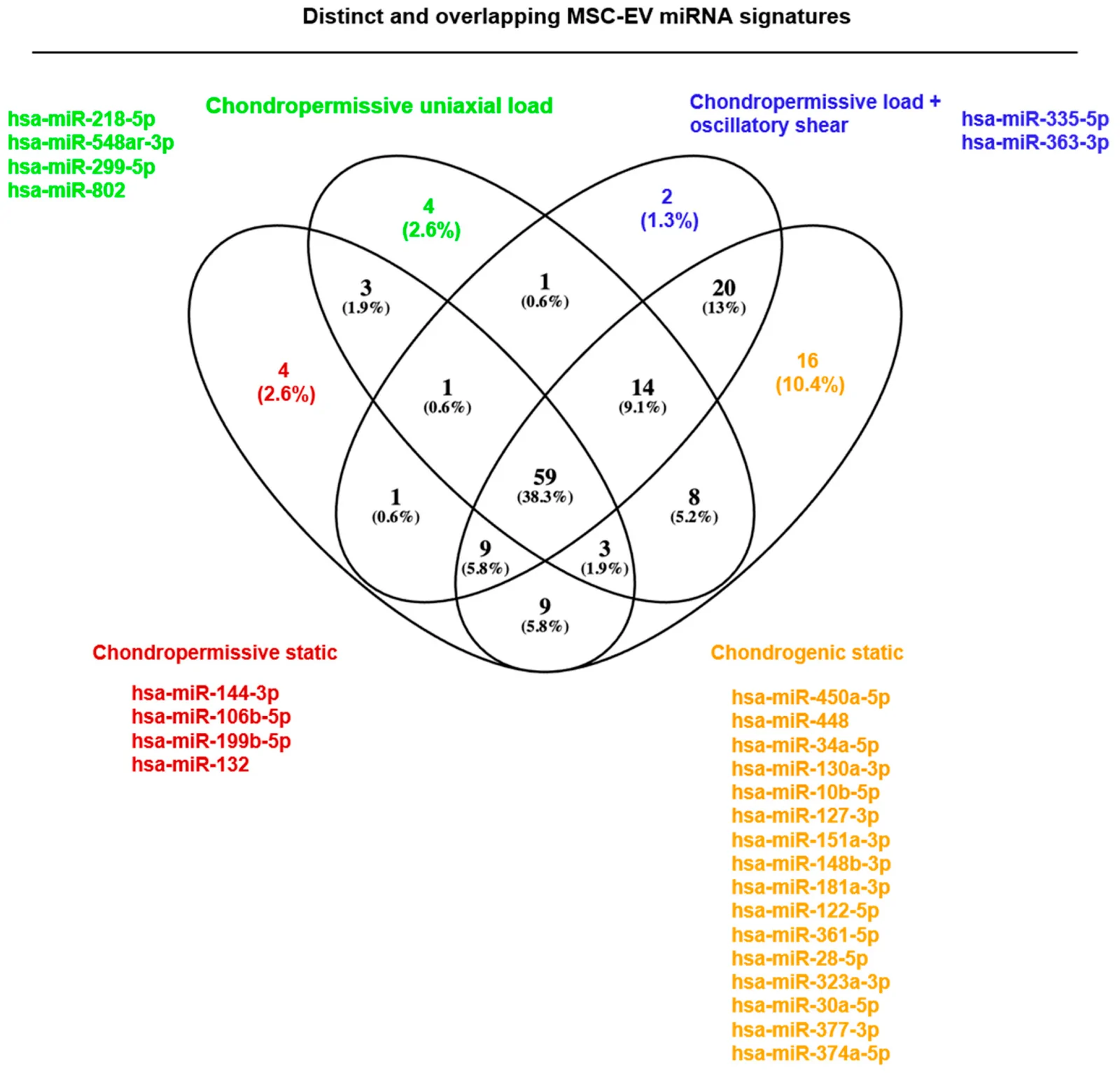
**Distinct and overlapping MSC-EV miRNA signatures.** All groups share a conserved regenerative/immunomodulatory core (59 miRNAs) enriched in immune signaling, NFκB/TLR pathways, and growth factor pathways including NGF, PDGF, FGFR, and EGFR. Condition-specific shifts are evident as chondrogenic static (CH) favored cell survival/proliferation pathways, CPL highlighted ECM/neurotrophin signaling, and CPLS showed strong TGF-β and MAPK enrichment.

**Figure 8:**
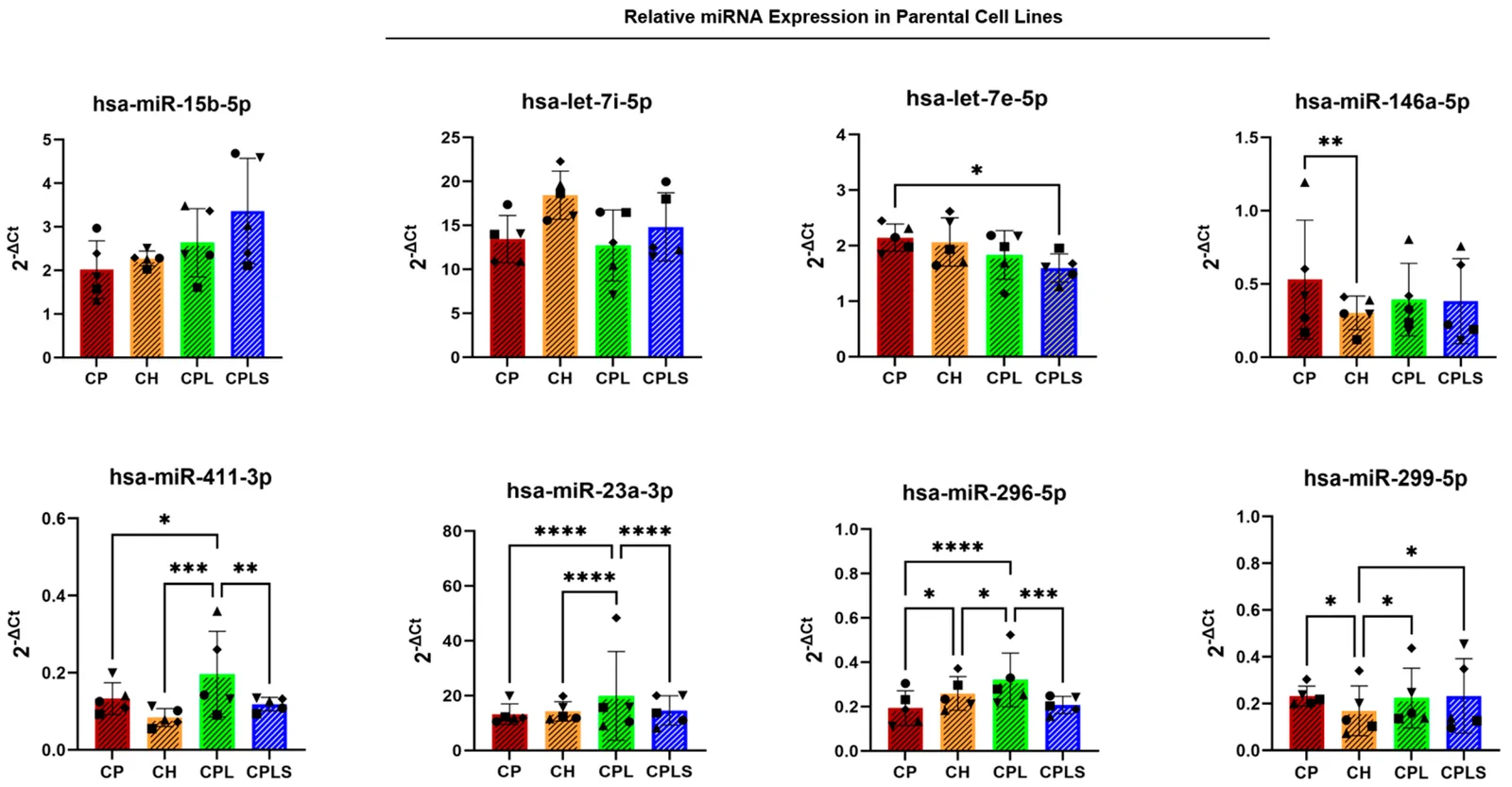
**Validation of key miRNA expression in parental MSCs across culture conditions.** This figure presents the relative expression of eight candidate miRNAs (hsa-let-7i-5p, hsa-miR-15b-5p, hsa-miR-146a-5p, hsa-let-7e-5p, hsa-miR-411-3p, hsa-miR-23a-3p, hsa-miR-299-5p, hsa-miR-296-5p) within the parental MSCs used for EV isolation across the four experimental groups (Red = chondropermissive static; green = chondropermissive uniaxial load; orange = chondrogenic static; and blue = chondropermissive uniaxial load with oscillatory shear). *n* = 5 donors, each represented by a unique symbol. Bars represent the average expression value, and error bars represent standard deviation. * *p* ≤ 0.05, ** *p* ≤ 0.01, *** *p* ≤ 0.001, **** *p* ≤ 0.0001.

**Figure 9:**
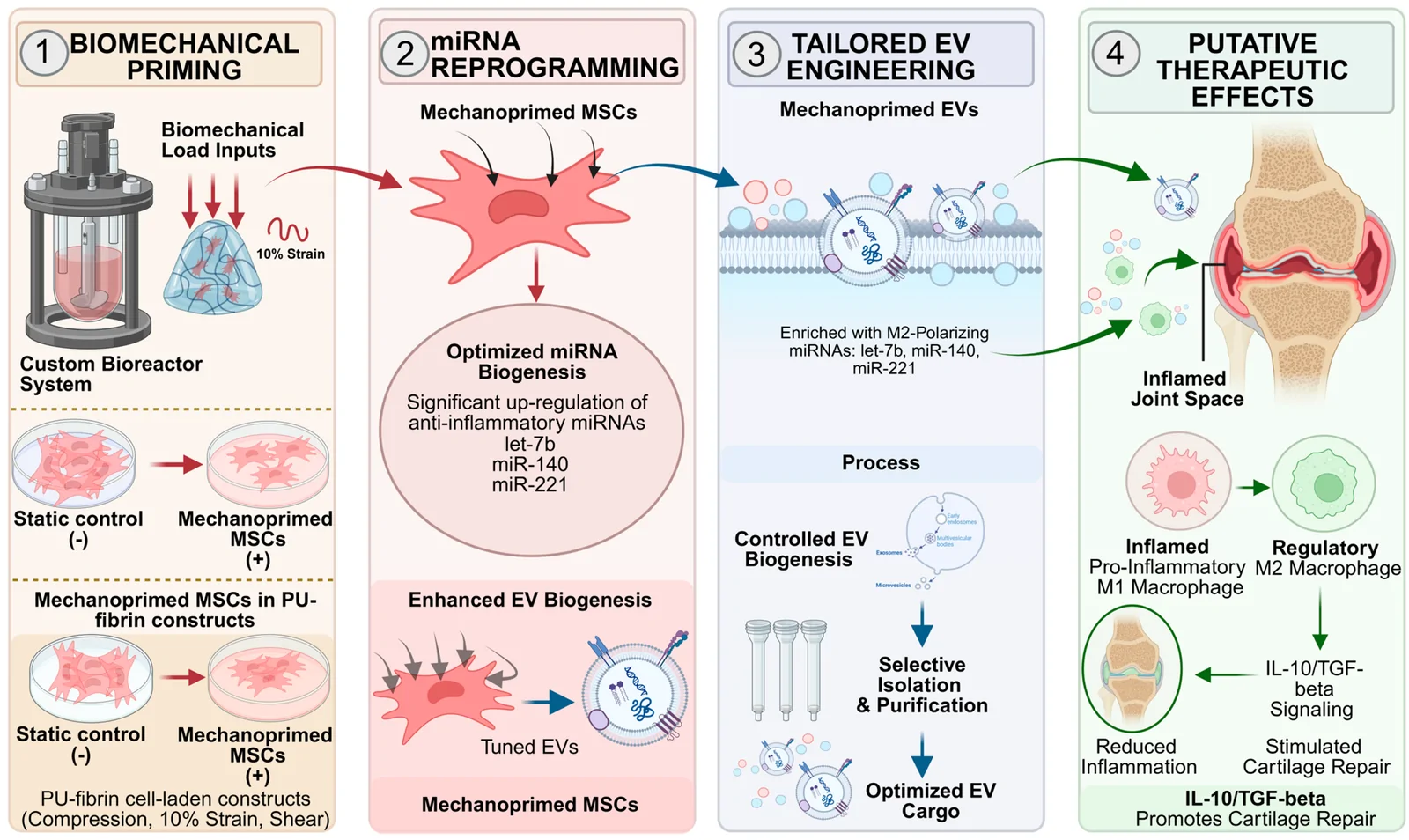
**Hypothetical blueprint for mechanobiological programming of MSC-EV therapeutics in osteoarthritis.** This visual synthesis illustrates a proposed hypothetical integrated workflow: (1) the multidimensional bioreactor inputs (mechanical vs. biochemical priming); (2) the induced ‘parental reprogramming’ of MSC mechanotransduction and miRNA signatures; (3) the resulting ‘tuned’ EV cargo (‘Mechanoprimed EVs’) with enriched anti-inflammatory potency; and (4) the putative downstream therapeutic cascade reducing inflammation and promoting cartilage repair in the inflammatory OA joint niche.

**Table 1:** Experimental timeline.

Day 0	Day 1	Day 2	Day 3	Day 4
Scaffold seeding	Loading	Medium change and Loading	Loading	Sample Harvesting

**Table 2:** **Number of EV-associated miRNAs predicted to target M2 macrophage-related regulatory domains across priming conditions**. Values represent the number of extracellular vesicle-associated microRNA species predicted to target each macrophage polarization- or immunomodulation-associated domain, including IL10 (anti-inflammatory marker), CD200R1 (immune suppression marker), CD163 (M2 macrophages polarization marker), MRC1 (M2 macrophages polarization marker), and NGF (M2 macrophages polarization support marker). The final column shows the total number of predicted M2-associated miRNA target hits for each priming condition. These counts reflect predicted miRNA-target associations and should not be interpreted as direct measurements of target gene or protein expression.

Group	IL10 Anti-Inflammatory	CD200R1 Immune Suppression	CD163 M2 Marker	MRC1 M2 Marker	NGF M2 Support	Total Predicted M2- Associated miRNA Hits
**CP**	8	8	3	3	7	29
**CH**	12	9	6	6	9	42
**CPL**	8	6	4	6	7	31
**CPLS**	10	9	6	6	9	40

## Data Availability

The original contributions presented in this study are included in the article. Further inquiries can be directed to the corresponding author.

## Abbreviations

α-MEM	alpha-minimum essential medium
ANOVA	analysis of variance
Bfgf	basic fibroblast growth factor
BM-MSC	bone marrow-derived mesenchymal stem/stromal cell
CD	cluster of differentiation
cDNA	complementary DNA
CH	chondrogenic static induction
CP	chondropermissive static culture
CPL	chondropermissive uniaxial compression
CPLS	chondropermissive uniaxial compression plus oscillatory shear
DEPC	diethylpyrocarbonate
DMEM	Dulbecco’s modified Eagle medium
DPBS	Dulbecco’s phosphate-buffered saline
ECM	extracellular matrix
EGFR	epidermal growth factor receptor
EV	extracellular vesicle
FBS	fetal bovine serum
FGFR	fibroblast growth factor receptor
GAPDH	glyceraldehyde 3-phosphate dehydrogenase
IL	Interleukin
IL10	interleukin 10
IFP	infrapatellar fat pad
MAPK	mitogen-activated protein kinase
miRNA	microRNA
MSC	mesenchymal stem/stromal cell
NF-κB	nuclear factor kappa B
NGF	nerve growth factor
NTA	nanoparticle tracking analysis
OA	Osteoarthritis
PDGF	platelet-derived growth factor
PEEK	polyether ether ketone
PU	Polyurethane
qPCR	quantitative polymerase chain reaction
RNA	ribonucleic acid
RT-qPCR	reverse transcription quantitative polymerase chain reaction
TGF-β	transforming growth factor beta
TLR	Toll-like receptor
TNF-α	tumor necrosis factor alpha
VEGFA	vascular endothelial growth factor A
Wnt	Wingless/integrated signaling pathway
